# Laparoscopic cholecystectomy in empyema of gall bladder: An experience at Liaquat University Hospital, Jamshoro, Pakistan

**DOI:** 10.4103/0972-9941.33273

**Published:** 2007

**Authors:** Arshad Malik, Abdul Aziz Laghari, K Altaf Hussain Talpur, Aisha Memon, Qasim Mallah, Jan Mohammad Memon

**Affiliations:** Department of Surgery, Liaquat University of Medical and Health Sciences, Jamshoro, Pakistan

**Keywords:** Empyema gall bladder, laparoscopic cholecystectomy, morbidity, safety

## Abstract

**Objective:**

To find out the safety profile of laparoscopic cholecystectomy in empyema of gallbladder.

**Background:**

Empyema of gall bladder is a severe form of acute cholecystitis with superadded suppuration. It has been considered a contraindication for the laparoscopic cholecystectomy (LC) because of fear of life-threatening complications. This study aimed to determine the safety and feasibility of LC in empyema of gallbladder.

**Materials and Methods:**

LC was attempted in 67 patients of empyema of gallbladder within 24h. However in few cases there was a delay because of reluctance for surgery or delay in giving consent etc. The procedure was performed by standard four-port technique with few changes made to facilitate dissection according to situation.

**Results:**

Between April 2003 to June 2006, 970 LC performed for gallstone disease at surgical unit-1 of LUMHS by the same surgical team. Among these, 67 (6.90%) patients were diagnosed to have empyema gall bladder. LC successfully completed in 54 (80.59%) patients. In 13 (19.40%) patients the procedure was converted to open cholecystectomy (OC) due to various operative difficulties of which the most serious injuries included bleeding from cystic artery (four cases), common bile duct injury (two cases) and duodenal injury in one case. Maximum operating time was up to 160 minutes (one case). Postoperative complications occurred in 10 (18.51%) successfully operated patients. Maximum patients (n=45, 83.33%) were discharged in 48-96 hours while three patients were discharged after two weeks.

**Conclusion:**

Laparoscopic cholecystectomy can be performed in empyema of gallbladder keeping in mind a slightly increased risk of complications even in the best hands. However, the experience of the surgeon plays a key role in the overall outcome.

## INTRODUCTION

The laparoscopic cholecystectomy (LC) has dramatically changed the outlook of patients with symptomatic gallstone disease. Empyema of the gallbladder is a potentially fatal complication of gallstones. It is characterized by suppuration superimposed on acute cholecystitis. The clinical presentation of this disease is often difficult to distinguish from acute cholecystitis.[1] Features suggesting diagnosis and seriousness of this disease are few.[2] It used to be a contraindication for LC because of fear of life-threatening complications.[3–7] It is also considered one of the commonest reasons for the conversion.[8] Increasing experience and technology in the field of laparoscopic surgery has brought a significant change and a number of studies have reported LC to be safe and effective option in acute cholecystitis and associated conditions like empyema of the gallbladder.[9–13] There can be various reasons and factors which can however, lead to conversion.[14] Obscured local anatomy, uncontrolled bleeding and damage to nearby vital structures are the common factors responsible for conversion.[15] Despite various encouraging reports, the role of laparoscopic surgery in such acute conditions is still under evaluation. This study aimed to find out safety and outcome of LC in empyema gallbladder.

## MATERIALS AND METHODS

This prospective experimental study was conducted in department of surgery (Unit-1), Liaquat University of Medical and Health Sciences Jamshoro, a teaching hospital in Sindh province of Pakistan, during April 2003 to June 2006. The LC was done by standard 4-port technique with few modifications depending upon the situation such as an additional port, percutaneous decompression of gallbladder by spinal needle. In case of thick pus the gallbladder was incised and the suction cannula directly introduced into gall bladder to aspirate pus. At times the suction cannula was also used to dissect the dense adhesions in the area of Calot's triangle. The thickened wall of the gallbladder was also incised to apply the graspers properly in cases where it was difficult to get hold of the thick, edematous gallbladder. Data of each patient was recorded on a data form including demographic details, operative findings, intraoperative complications, post operative complications and duration of hospitalization. The results were analyzed on SPSS version 10. A well-informed written consent was taken from each patient prior to surgery.

### Inclusion criteria

All patients with clinical, sonological and biochemical evidence of cholelithiasis with empyema were included in the study regardless of age and gender.

### Exclusion criteria

Patients with major medical problems in which pneumoperitoneum was thought to be unsafe and those with overwhelming sepsis were excluded from the study.

## RESULTS

Nine hundred and seventy LC were performed for gallstone disease, of which 319 (32.88%) patients were found to have complicated gallstone disease. Empyema gallbladder forms a major component in the complicated gallstone disease and account for 21.00% of the complicated gallstone disease in this series. Of the total laparoscopic cholecystectomies, 67 (6.90%) patients of empyema of gallbladder were identified and included in the study population. The diagnosis of empyema gallbladder was established on the basis of such findings as tender, palpable gallbladder, leucocytosis > 12000 cc, ultrasound findings and per-operative aspiration of frank pus from edematous gallbladder. The age ranged from 21 years to 71 with a mean of 40.82 years. There were nine males and 58 females in the study population with male to female ratio of 1:6.4. The main diagnostic criteria is shown in Table 1 A and B. Preoperative diagnosis was established in 42 (62.68%) patients while remaining 25 (37.31%) patients were identified during surgery. All of these patients were operated laparoscopically within 24h of the admission. Fifty-four (80.59%) LC were completed successfully while in 13 (19.40%) patients the procedure was converted to OC due to various reasons as shown in Table 2. Conversion was found more common in those who had history of repeated attacks of acute cholecystitis in the past. Total operating time ranged from 60-160 minutes with a mean of 87 minutes. Maximum patients (70.14%) were operated between 60-90 minutes. Operative complications of varying degrees and severity occurred in 12 (22.22%) out of 54 successfully operated cases [Table 3A]. The overall rate of postoperative complications was 18.51% in successfully completed LC and 61.53% in the converted population [Table 3B]. Majority of the patients (83.33%) with successful LC were discharged with in 48-96h. In six patients (11.11%), the stay in hospital was extended to seven days. The remaining three (5.55%) patients were discharged in two weeks time. The average hospital stay in patients converted to open operation was 10 days. Wound sepsis was found to be more common in this group, contributing to an increased overall morbidity.

**Table 1A:** Diagnosis. Clinical features

	No. of patients
Pain in right hypochondrium	67 (100)
Fever	51 (76.11)
Vomiting	19 (28.35)
Palpable gallbladder	39 (58.20)

Values in parentheses are percentages

**Table 1B:** Diagnosis. Ultrasound findings

Keep A and B together	No. of patients
Distended gallbladder	52 (77.61)
Thickened wall of gallbladder	56 (83.58)
Intraluminal sludge or stones	64 (95.52)
Pericholecystic fluid accumulation	39 (58.20)

Values in parentheses are percentages

**Table 2:** Reasons for conversion to open cholecystectomy.

	No. of patients
Totally obscured anatomy in the Calot's triangle	6 (46.15)
Bleeding	4 (30.76)
Common bile duct injury	2 (15.38)
Duodenal perforation	1 (7.69)

Values in parentheses are percentages

**Table 3A:** Operative complications

Complication	No. of cases	%
Perforation of gallbladder	04	7.40
Minor trauma to common bile duct	03	5.55
Bleeding	04	7.40
Duodenal perforation	01	1.85

**Table 3B:** Postoperative complications

Complication	Successfully operated (n=54)	Converted (n=13)
Port site/wound infection	3 (5.55)	3 (23.07)
Bile leak	2 (3.70)	1 (7.69)
Intra-abdominal collection	3 (5.55)	2 (15.38)
Chest infection	2 (3.70)	2 (15.38)
Total	10/54 (18.51)	8/13 (61.53)

Values in parentheses are percentages. n= 13/54

## DISCUSSION

LC has become a preferred and acceptable choice even in the most difficult situations associated with complicated gallstone disease.[16–18] The earlier arguments[19] as to its safety and efficacyare being answered by a number of encouraging reports[9–13] and more and more laparoscopic surgeons are persuaded to perform LC in acute cholecystitis as suggested by Hunter[20] “to get it while its Hot”. Very few reports have specifically assessed safety of LC in empyema of the gallbladder. This study presents the details of 67 LC performed in empyema gallbladder within 24h of the admission to asses the safety and suitability of laparoscopic approach in this condition. The difficulties that we encountered in dissection in the area of Calot's triangle are more or less the same as mentioned by other similar studies.[14] The overall conversion rate in this study (19.40%) is consistent with other reports.[7,21] History of recurrent acute cholecystitis and an undue delay in the surgery are the main contributing factors for conversion in this study, a finding consistent with other similar studies.[14,22–24] The nature of the study population must also be known as suggested by Gouma.[25] The study population in this report is mainly from poor socio-economic background, coming from remote areas of rural Sindh province of Pakistan. There is a general reluctance for surgery in these patients because of economical reasons in addition to general fear for surgery. Their presentation is therefore delayed and operation is technically difficult due to fibrosis and firm adhesions. These are the common factors producing distortion of local anatomy.[26,27] This has been the main factor in conversions in this series with an additional contribution of our standing on the learning curve. The conversion rate can be significantly reduced by patience, clear display and identification of the anatomy of Calot's triangle before cutting or applying clips. The dissection should proceed with extreme caution and gentle separation of the adhesion should be done. Duodenum should be identified and be gently pushed down to avoid injury. The use of diathermy should be minimal and threshold for conversion should be kept low to ensure patients safety. We decompressed the distended gallbladder before proceeding to Calot's triangle to facilitate dissection. Tseng *et al.*[28] have also favored this procedure to make surgery safe and easier. Another way of handling such life threatening situations is to perform subtotal cholecystectomy after removal of all the stones to ensure safety of patients life instead of continuing dissection in the frozen Calot's triangle with totally obscured anatomy. The rate of major complications is not significant in current study as to preclude the laparoscopic approach in this condition but there should always be a word of caution while operating on such difficult conditions. This is consistent with the findings of Hobbs *et al.*[29] claiming that increased risk of complications with LC has stabilized. However few cases of major cystic artery bleed and duodenal perforation occurred and we had to resort to open technique considering safety of the patients. The cystic artery bleed was initially attempted to be controlled by temponade and gauze pressure, failing which we converted the cases and bleeding controlled. The duodenal perforation was identified then and there and the operation was converted with subsequent primary closure of duodenum. There is always a risk of common bile duct (CBD) injury if the operating surgeon is impatient and anatomy of the field is not clearly displayed before clipping and cutting. Undue use of diathermy is also a major factor in causing CBD injury and should be avoided in the area of Calot's triangle. The operative trauma occurred to CBD in two cases of which one was managed by placing a T-tube and in the other we performed primary repair of the CBD. Both did well in the postoperative period. The total hospital stay in the converted population was prolonged with an average of 10 days. This is, however, contrary to the finding of Johansson *et al.*[30] claiming that conversion did not prolong the postoperative hospital stay in the study population.

LC in empyema has shown less morbidity and no mortality in our study. The analysis of our study and literature review has shown that this procedure was associated with less intraoperative blood loss, shorter hospital stay, less wound infection and less postoperative pain.

## CONCLUSION

LC is a safe and acceptable option in empyema of gallbladder. There are, however, significant technical difficulties due to edema, adhesions and distorted anatomy in the area of Calot's triangle. The experience of the surgeon plays a vital role. We recommend that patient's safety should be given priority and threshold for conversion should be kept lower and sub-total cholecystectomy may be considered where ever dissection is found to be dangerous.
